# Supplementary material for: HIVprotI: an integrated web based platform for prediction and design of HIV proteins inhibitors
Source: J Cheminform. 2018 Mar 9;10:12. doi: 10.1186/s13321-018-0266-y (PMC5845081; doi:10.1186/s13321-018-0266-y)
Supplement: Supplementary file 2 — Additional file 2. Source code of HIVProtI web server. [file 13321_2018_266_MOESM2_ESM.zip › HIVprotI_Source-code/hivprotI/weka-3-6-6/doc/weka/core/mathematicalexpression/sym.html]

sym


---


|  |  |  |
| --- | --- | --- |
| **Overview** \| **Package** \| **Class** \| **Tree** \| **Deprecated** \| **Index** \| **Help** \| **Weka's home** | |  |
| **PREV CLASS**   NEXT CLASS | **FRAMES**    **NO FRAMES**     **All Classes** |  |
| SUMMARY: NESTED \| FIELD \| CONSTR \| METHOD | DETAIL: FIELD \| CONSTR \| METHOD |  |


---


## weka.core.mathematicalexpression Interface sym

---

``` public interface sym ```

CUP generated interface containing symbol constants.

---

| **Field Summary** | |
| --- | --- |
| `static int` | `ABS` |
| `static int` | `AND` |
| `static int` | `BOOLEAN` |
| `static int` | `CEIL` |
| `static int` | `COMMA` |
| `static int` | `COS` |
| `static int` | `DIVISION` |
| `static int` | `EOF` |
| `static int` | `EQ` |
| `static int` | `error` |
| `static int` | `EXP` |
| `static int` | `FALSE` |
| `static int` | `FLOOR` |
| `static int` | `GE` |
| `static int` | `GT` |
| `static int` | `IFELSE` |
| `static int` | `LE` |
| `static int` | `LOG` |
| `static int` | `LPAREN` |
| `static int` | `LT` |
| `static int` | `MINUS` |
| `static int` | `NOT` |
| `static int` | `NUMBER` |
| `static int` | `OR` |
| `static int` | `PLUS` |
| `static int` | `POW` |
| `static int` | `RINT` |
| `static int` | `RPAREN` |
| `static int` | `SIN` |
| `static int` | `SQRT` |
| `static int` | `TAN` |
| `static int` | `TIMES` |
| `static int` | `TRUE` |
| `static int` | `VARIABLE` |

**Field Detail**

### TIMES

```
static final int TIMES
```

**See Also:**: Constant Field Values

---


### AND

```
static final int AND
```

**See Also:**: Constant Field Values

---


### IFELSE

```
static final int IFELSE
```

**See Also:**: Constant Field Values

---


### LT

```
static final int LT
```

**See Also:**: Constant Field Values

---


### SIN

```
static final int SIN
```

**See Also:**: Constant Field Values

---


### PLUS

```
static final int PLUS
```

**See Also:**: Constant Field Values

---


### OR

```
static final int OR
```

**See Also:**: Constant Field Values

---


### RPAREN

```
static final int RPAREN
```

**See Also:**: Constant Field Values

---


### COS

```
static final int COS
```

**See Also:**: Constant Field Values

---


### DIVISION

```
static final int DIVISION
```

**See Also:**: Constant Field Values

---


### NOT

```
static final int NOT
```

**See Also:**: Constant Field Values

---


### POW

```
static final int POW
```

**See Also:**: Constant Field Values

---


### SQRT

```
static final int SQRT
```

**See Also:**: Constant Field Values

---


### VARIABLE

```
static final int VARIABLE
```

**See Also:**: Constant Field Values

---


### TRUE

```
static final int TRUE
```

**See Also:**: Constant Field Values

---


### FLOOR

```
static final int FLOOR
```

**See Also:**: Constant Field Values

---


### RINT

```
static final int RINT
```

**See Also:**: Constant Field Values

---


### GT

```
static final int GT
```

**See Also:**: Constant Field Values

---


### CEIL

```
static final int CEIL
```

**See Also:**: Constant Field Values

---


### LPAREN

```
static final int LPAREN
```

**See Also:**: Constant Field Values

---


### LE

```
static final int LE
```

**See Also:**: Constant Field Values

---


### LOG

```
static final int LOG
```

**See Also:**: Constant Field Values

---


### EXP

```
static final int EXP
```

**See Also:**: Constant Field Values

---


### BOOLEAN

```
static final int BOOLEAN
```

**See Also:**: Constant Field Values

---


### COMMA

```
static final int COMMA
```

**See Also:**: Constant Field Values

---


### NUMBER

```
static final int NUMBER
```

**See Also:**: Constant Field Values

---


### EOF

```
static final int EOF
```

**See Also:**: Constant Field Values

---


### ABS

```
static final int ABS
```

**See Also:**: Constant Field Values

---


### GE

```
static final int GE
```

**See Also:**: Constant Field Values

---


### FALSE

```
static final int FALSE
```

**See Also:**: Constant Field Values

---


### MINUS

```
static final int MINUS
```

**See Also:**: Constant Field Values

---


### error

```
static final int error
```

**See Also:**: Constant Field Values

---


### EQ

```
static final int EQ
```

**See Also:**: Constant Field Values

---


### TAN

```
static final int TAN
```

**See Also:**: Constant Field Values


---


|  |  |  |
| --- | --- | --- |
| **Overview** \| **Package** \| **Class** \| **Tree** \| **Deprecated** \| **Index** \| **Help** \| **Weka's home** | |  |
| **PREV CLASS**   NEXT CLASS | **FRAMES**    **NO FRAMES**     **All Classes** |  |
| SUMMARY: NESTED \| FIELD \| CONSTR \| METHOD | DETAIL: FIELD \| CONSTR \| METHOD |  |


---
